# In Situ Hydrogel Growth on Flame-Sprayed Hydroxyapatite (HA)/TiO_2_-Coated Stainless Steel via TiO_2_-Photoinitiated Polymerization

**DOI:** 10.3390/gels11100837

**Published:** 2025-10-18

**Authors:** Komsanti Chokethawai, Nattawit Yutimit, Burin Boonsri, Parkpoom Jarupoom, Ketmanee Muangchan, Sahadsawat Tonkaew, Pongpen Kaewdee, Sujitra Tandorn, Chamnan Randorn

**Affiliations:** 1 Department of Physics and Materials Science, Faculty of Science, Chiang Mai University, Chiang Mai 50200, Thailand; komsanti.chokethawai@cmu.ac.th (K.C.); nattawit.yutimit@gmail.com (N.Y.); 2 Faculty of Veterinary Medicine, Chiang Mai University, Chiang Mai 50200, Thailand; burin.b@cmu.ac.th; 3 Department of Industrial Engineering, Faculty of Engineering, Rajamangala University of Technology Lanna (RMUTL), Chiang Mai 50300, Thailand; noteparkpoom@gmail.com; 4 Materials and Medical Innovation Research Unit, Faculty of Engineering, Rajamangala University of Technology Lanna (RMUTL), Chiang Mai 50300, Thailand; 5 Department of Chemistry, Faculty of Science, Chiang Mai University, Chiang Mai 50200, Thailand; ketmanee.mu@gmail.com (K.M.); sahadsawat_to@cmu.ac.th (S.T.); pongpen.k@cmu.ac.th (P.K.); sujitra.tandorn@cmu.ac.th (S.T.); 6 Office of Research Administration, Chiang Mai University, Chiang Mai 50200, Thailand; 7 Center of Excellence in Materials Science and Technology, Chiang Mai University, Chiang Mai 50200, Thailand

**Keywords:** hydroxyapatite, coating, hydrogel, photoinitiation, implant

## Abstract

Hydroxyapatite (HA) coatings improve implant bioactivity but suffer from brittleness and limited functionality. Here, we report a hybrid coating strategy combining flame-sprayed HA/TiO_2_ with in situ hydrogel growth. TiO_2_ incorporated into the HA matrix acted as a photocatalytic initiator for acrylamide polymerization under UV. Unlike conventional hydrogel coatings that require added photoinitiators or separate surface modification steps, TiO_2_ incorporated into the HA layer serves as a built-in photocatalytic initiator, enabling direct polymerization of acrylamide monomers on the sprayed surface. The resulting HA/TiO_2_–hydrogel coatings exhibited a continuous hydrogel layer with intimate contact to the ceramic surface, as evidenced by SEM cross-sections and elemental mapping. The HA/TiO_2_ 1% coating produced a continuous coverage in close contact with the surface, while excessive TiO_2_(5%) led to uncontrolled hydrogel growth and partial coating failure. SEM cross-sections revealed a dense, well-adhered coating with homogeneously distributed Ca, P, O, and finely dispersed Ti. Upon immersion in simulated body fluid (SBF), submicron globular deposits progressively developed on the coating surface. EDS showed an increase in Ca/P ratio from ~1.66 (as-sprayed) to ~1.92 (14 days). These findings highlight a straightforward approach for combining flame-sprayed ceramics with photocatalytic hydrogel growth, providing a practical route toward multifunctional implant surface modification.

## 1. Introduction

Bone damage resulting from trauma, infection, congenital malformations, or degenerative diseases often requires the use of load-bearing implants to restore function and structural integrity. Metallic implants remain the materials of choice for orthopedic and dental applications because of their high strength and toughness. Titanium and its alloys are widely regarded as the standard implant materials due to their excellent corrosion resistance and biocompatibility [1,2,3,4]. However, titanium implants have notable drawbacks: their high cost limits accessibility, their relatively low wear resistance can shorten longevity in articulating joints, and hypersensitivity or mechanical mismatch may lead to stress shielding and impaired bone remodeling.

Stainless steel (SS), particularly 316L, remains an attractive and cost-effective alternative for load-bearing implants, though it offers lower corrosion resistance and biocompatibility than titanium alloys. A major limitation of stainless steel, however, is its intrinsic bioinertness, which can result in fibrous encapsulation, aseptic loosening, and eventual implant failure [5]. Thus, surface modification strategies are essential to enhance the biological response of stainless steel implants while preserving their mechanical robustness. Among these, surface coatings are a particularly practical and effective approach.

Hydroxyapatite (HA; Ca_10_(PO_4_)_6_(OH)_2_) has long been explored as a bioactive coating due to its chemical similarity to bone mineral. HA promotes osseointegration by supporting osteoblast adhesion and controlled ion release [6,7,8,9,10]. Several deposition methods have been developed: wet-chemical routes (sol–gel, hydrothermal) provide good chemical control but suffer from poor adhesion; plasma spraying produces thick coatings but risks phase degradation and high cost; and HVOF spraying improves phase retention but requires complex equipment [11,12,13]. Flame spraying, in contrast, is a simpler, scalable, and cost-effective method that yields coatings with controllable phase composition and reliable adhesion [14,15]. However, the smooth and compact surfaces produced often limit protein adsorption, cell attachment, and bone integration.

Hydrogels represent another promising coating strategy. As three-dimensional, water-rich polymer networks, they mimic the extracellular matrix and offer versatility for biomedical applications. Hydrogel coatings can be fabricated by dip coating, spin coating, electrospray, or chemical vapor deposition, and biopolymers such as chitosan, alginate, gelatin, and polyacrylamide have been shown to enhance osteoblast activity and provide antibacterial functionality [16].

Since HA coatings are bioactive but brittle with poor adhesion, and hydrogel coatings are cell-supportive but mechanically weak, combining them offers complementary advantages. HA–hydrogel hybrids integrate mineral-mediated bonding with the flexibility and ion-exchange capacity of hydrogels, producing multifunctional coatings with improved stability and biological performance. For example, hydrogel/HA nanocomposites in PCL/HEMA matrices improved adhesion and osteoblast activity [17]; dip-coated PVA/HA composites achieved tunable HA exposure for ligament anchoring [18]; and HA–MXene/PVA/PAA/PDA hydrogels fabricated by sol–gel and spin coating enhanced anticorrosion properties in SBF [19]. More recently, a wide range of hydrogel-based coatings have been explored for metallic implants to further enhance integration and multifunctionality. For example, a hybrid system in which liposomes are embedded within a gelatin methacryloyl (GelMA) hydrogel has been applied to titanium, promoting osteogenesis [20]. Similarly, biodegradable sodium alginate and carboxymethyl chitosan (SA/CMCS) hydrogels coated onto magnesium alloys have been shown to facilitate cell growth and proliferation [21]. Another approach has demonstrated that hydrogel coatings can enhance bone–titanium integration by mediating sequential M1/M2 polarization of interfacial macrophages [22]. These advances highlight the versatility of hydrogel coatings but also reveal that many rely on dip-coating or solution-based methods, which may limit adhesion strength and long-term stability under physiological conditions.

Despite these advances, challenges remain for HA–hydrogel composites. Many reported coatings are produced by dip-coating or sol–gel approaches, which can result in hydrogel layers that are loosely attached and prone to delamination in wet environments. At the same time, flame spraying is an industrially scalable method that yields adherent HA/TiO_2_ coatings, but these surfaces are relatively smooth and lack sufficient biological functionality. This contrast highlights a gap in strategies that can integrate the coating stability of flame spraying with the biological advantages of hydrogels. In this work, we explore a proof-of-concept approach in which TiO_2_ incorporated into a flame-sprayed HA/TiO_2_ layer is used as a built-in photocatalyst to initiate in situ polymerization of a polyacrylamide hydrogel. Polyacrylamide-based hydrogels have been reported to improve the in vivo performance of implanted devices, with coatings showing reduced inflammation and extended functional lifetime, supporting their use as acceptable biocompatible materials in certain applications [23]. This approach enables direct hydrogel growth on the ceramic coating and allows us to investigate fabrication feasibility and mineralization behavior in simulated body fluid.

## 2. Results and Discussion

**Figure 1:**
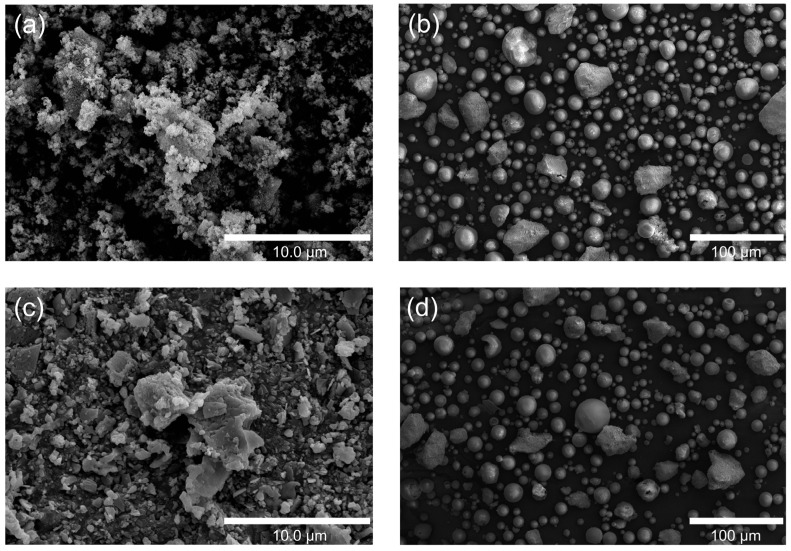
SEM images of HA/TiO_2_–1%: (**a**) as-sprayed powders and (**b**) in-flight particles, and HA/TiO_2_–5%: (**c**) as-sprayed powders and (**d**) in-flight particles.

The SEM images compare the morphology of HA/TiO_2_ powders and their in-flight counterparts quenched in a water bath during flame spraying. The as-prepared HA/TiO_2_–1% powders (Figure 1a) appear as irregular, agglomerated clusters with rough surfaces, while the corresponding in-flight particles (Figure 1b) are predominantly spherical with smooth surfaces, indicating effective melting and rapid solidification during spraying. In contrast, the HA/TiO_2_–5% powders (Figure 1c) show larger aggregates and more angular fragments, and their in-flight particles (Figure 1d) exhibit a higher fraction of partially melted or irregular morphologies. These results suggest that although flame spraying promotes spheroidization of HA/TiO_2_ powders, higher TiO_2_ content reduces melting efficiency, leading to less uniform particle morphology that may affect subsequent coating microstructure and performance.

**Figure 2:**
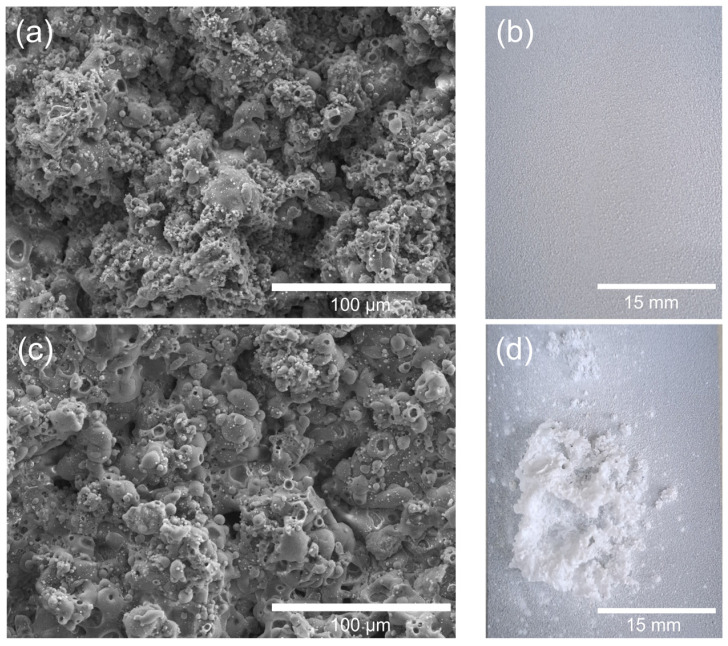
Effect of TiO_2_ content in flame-sprayed HA/TiO_2_ coatings on hydrogel growth: (**a**) SEM image of HA/TiO_2_–1% before hydrogel growth, (**b**) corresponding visual image after hydrogel growth, (**c**) SEM image of HA/TiO_2_–5% before hydrogel growth, and (**d**) corresponding visual image after hydrogel growth.

The SEM images of the as-sprayed coatings (Figure 2a,c) display a rough, particulate surface with interconnected micro- and submicron-sized pores, typical of flame-sprayed HA/TiO_2_ deposits. Such morphology is beneficial, as it increases surface area and provides anchoring sites for subsequent hydrogel attachment. After immersion in acrylamide solution and UV exposure, the visual appearance revealed clear differences in hydrogel growth. For HA/TiO_2_–1% (Figure 1b), the hydrogel layer formed uniformly across the surface, preserving coating integrity. In contrast, HA/TiO_2_–5% (Figure 2d) showed excessive hydrogel accumulation, which generated swelling stress and led to partial peeling of the coating.

This outcome highlights the dual role of TiO_2_ incorporation. While TiO_2_ acts as an efficient photocatalytic initiator for hydrogel polymerization, excessive TiO_2_ content (5%) accelerates the reaction and promotes uncontrolled hydrogel growth, compromising the mechanical stability of the HA/TiO_2_ coating. A lower TiO_2_ content (1%) provides a more balanced initiation effect, yielding a uniform and adherent hydrogel layer.

**Figure 3:**
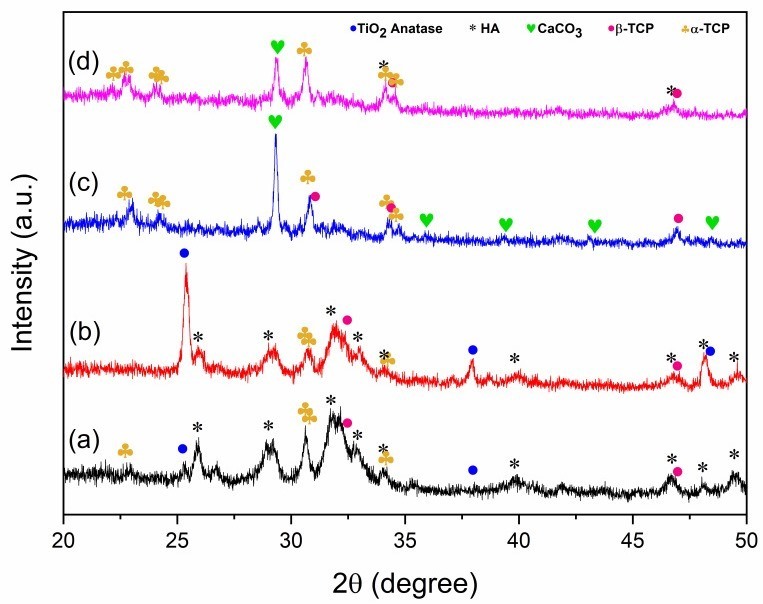
XRD patterns of HA/TiO_2_: (**a**) 1 wt% TiO_2_ powder, (**b**) 5 wt% TiO_2_ powder, and after flame-spray coating (**c**) HA/TiO_2_–1% and (**d**) HA/TiO_2_–5%.

The XRD patterns of HA/TiO_2_ powders and their corresponding flame-sprayed coatings are shown in Figure 3. For the precursor powders (Figure 3a,b), characteristic peaks of HA were clearly detected together with α-TCP and β-TCP phases, indicating that the synthesized powders consist of a multiphase calcium phosphate mixture rather than a single-phase HA. The incorporation of TiO_2_ was confirmed by anatase reflections, which were more pronounced in the 5% TiO_2_ sample. After flame spraying (Figure 3c,d), the coatings exhibited decreased HA peak intensity and the emergence of CaCO_3_ reflections, consistent with phase transformation and carbonation during the high-temperature spraying process. It is plausible that in flame-sprayed HA/TiO_2_ coatings, localized thermal fluctuations induced by TiO_2_ promote HA destabilization to CaO, which subsequently carbonates to CaCO_3_ upon cooling. This interpretation is further supported by our previous work on flame-sprayed nano-HA coatings without TiO_2_, in which no CaCO_3_ formation was observed [24]. The contrast suggests that TiO_2_ plays a critical role in facilitating CaCO_3_ generation during the coating process. Although TiO_2_ incorporation facilitates partial HA transformation, CaCO_3_ should not be regarded as undesirable. While the higher solubility of CaCO_3_ may compromise the long-term stability and mechanical integrity of the coating, it can also promote rapid Ca^2+^ release and enhance apatite nucleation, resulting in more homogeneous mineralization during SBF immersion. Therefore, the role of CaCO_3_ in HA/TiO_2_ coatings should be considered in a balanced manner, recognizing both its potential to improve short-term bioactivity and its possible drawbacks for long-term durability. Notably, owing to its biocompatibility, biodegradability, and higher solubility, CaCO_3_ has been shown to accelerate ionic release and stimulate bone formation, consistent with previous reports on CaCO_3_–CaP biphasic systems [25,26,27]. Both archeological evidence and engineered biomaterials reinforced with CaCO_3_ [28] further highlight its osteoconductivity, supporting CaCO_3_ as a beneficial component that contributes to controlled degradation and osteointegration in HA/TiO_2_ coatings.

**Figure 4:**
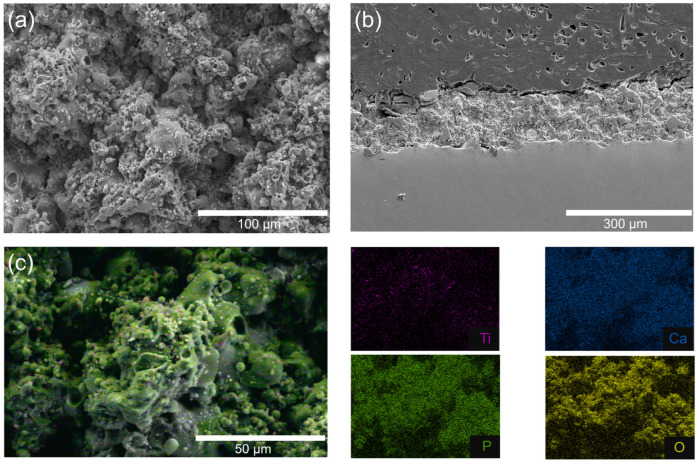
SEM images of HA/TiO_2_–1% coating: (**a**) top surface morphology, (**b**) cross-sectional view, and (**c**) corresponding EDS elemental mapping.

Figure 4 presents the structural and compositional characterization of the flame-sprayed HA/TiO_2_–1% coating, which was selected as the optimized condition for subsequent hydrogel growth studies. The surface SEM micrograph (Figure 4a) shows a rough and granular morphology, typical of flame-sprayed deposits, which provides high surface area and anchoring sites for hydrogel attachment. The cross-sectional SEM image (Figure 4b) reveals a dense, well-adhered coating layer with a thickness of approximately 150–300 µm, strongly bonded to the stainless steel substrate without visible delamination or cracks. Elemental mapping further confirmed the homogeneous distribution of Ti, Ca, P, and O throughout the coating. The Ti Kα map shows a fine dispersion of TiO_2_ within the HA matrix, consistent with its role as a photocatalytic initiator. The merged EDS map (Figure 4c) demonstrates that these elements are well interspersed, confirming compositional homogeneity at the microscale. These findings indicate that flame spraying successfully produced a robust HA/TiO_2_–1% composite coating with uniform elemental distribution and good adhesion to the substrate. The flame-spraying process also demonstrated good reproducibility, as confirmed by cross-sectional SEM images shown in Figure S1. The coating thickness obtained from different trials remained consistent, with a relative standard deviation (%RSD) of less than 10% for each measurement. Statistical analysis using one-way ANOVA showed no significant difference between groups (*p* > 0.05), confirming uniform deposition and stable process control. This reproducibility supports the reliability of the coating method and ensures that the subsequent hydrogel growth and SBF experiments were conducted on comparable substrates.

**Figure 5:**
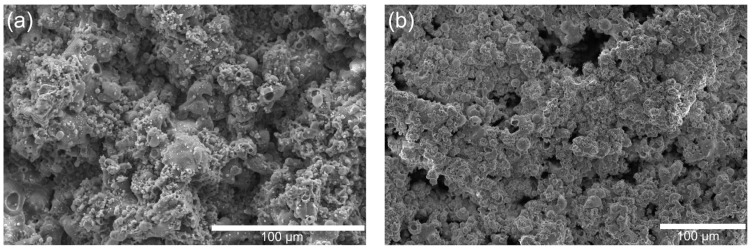
SEM micrographs of HA/TiO_2_-1% coatings (**a**) after flame spray deposition and (**b**) after in situ hydrogel growth.

The surface morphology of the as-sprayed HA/TiO_2_-1% coatings before and after hydrogel growth is shown in Figure 5. Prior to hydrogel deposition (Figure 5a), the coating displayed a compact microspherical structure, with relatively smooth surfaces and limited surface porosity. After hydrogel growth (Figure 5b), however, the surface features changed significantly. The hydrogel layer introduced additional roughness and porosity, with numerous nodular deposits decorating the microspheres and filling interparticle gaps. This transformation confirms the successful in situ polymerization of the hydrogel on the HA/TiO_2_ coating. FTIR analysis was additionally performed to identify the functional groups present in both the HA/TiO_2_ powder and the hydrogel/HA/TiO_2_ coating. The corresponding FTIR spectra are shown in Figure S2a,b, respectively. In the FTIR spectrum of HA/TiO_2_ powder, characteristic peaks of PO_4_^3−^ groups in hydroxyapatite (HA) were observed at 1094, 1015, 960, and 555 cm^−1^ [29,30,31], while peaks at approximately 425, 450, and 720 cm^−1^ were attributed to the vibrations of Ti–O and Ti–O–Ti bonds in TiO_2_ [32,33,34]. In the FTIR spectrum of the hydrogel/HA/TiO_2_ coating, peaks at 3345 and 3179 cm^−1^ were assigned to the N–H stretching vibrations of –NH_2_ groups. Additionally, characteristic peaks at 1650, 1606, and 1455 cm^−1^ corresponded to the C=O stretching, N–H bending, and C–N stretching vibrations of the amide group, respectively [35,36,37]. The presence of these amide-related peaks confirms the formation of polyacrylamide within the hydrogel matrix, indicating successful polymerization of acrylamide, with TiO_2_ acting as a photo-initiator under UV irradiation. However, after coating with the hydrogel, the characteristic peaks of PO_4_^3−^ and TiO_2_ were difficult to clearly identify, likely due to attenuation of the inorganic signals caused by coverage of HA/TiO_2_ by the hydrogel layer.

In addition, as shown in Figure S3, SEM–EDS analysis of the initial stage of in situ photopolymerization (after 1 h of irradiation) provides clear evidence of hydrogel growth across the HA/TiO_2_ surface. Two distinct surface morphologies can be observed: rod-like or flake-like structures and smoother regions distributed throughout the coating. The area marked on the rod-like structure shows the presence of nitrogen (13.7 wt%), indicating the incorporation of acrylamide functional groups from the polyacrylamide hydrogel. In contrast, the EDS spectrum obtained from the underlying coating region shows no detectable nitrogen, representing the HA/TiO_2_ surface beneath the hydrogel layer. These findings confirm that the hydrogel component formed both over and within the HA/TiO_2_ surface through photocatalytically initiated polymerization.

The swelling capacity of the hydrogel/HA/TiO_2_ composite was also evaluated in terms of the percentage swelling ratio. The experiment was conducted by monitoring change in the initial weight of the dry material before and after water absorption at various time intervals. As shown in Figure S4, the hydrogel/HA/TiO_2_ composite exhibited a gradual increase in swelling, reaching an equilibrium value of 110.72% after approximately 120 min. The equilibrium swelling ratio of the hydrogel/HA/TiO_2_ composite (~110%) is lower than that typically reported for bulk polyacrylamide hydrogels (300–1000%). This reduced swelling can be attributed to the limited amount of TiO_2_ in the coating, which leads to a smaller quantity of hydrogel formed during the photoinitiated polymerization process. Consequently, the hydrogel network is relatively thin and constrained by the underlying HA/TiO_2_ matrix, restricting its expansion in water.

The hydrogel introduces hydrated domains and ion-binding functional groups that enhance the effective surface area and generate micro–nanoscale porosity. These features are expected to facilitate the uptake of Ca^2+^ and phosphate ions from SBF and to promote uniform nucleation of apatite during immersion tests.

**Figure 6:**
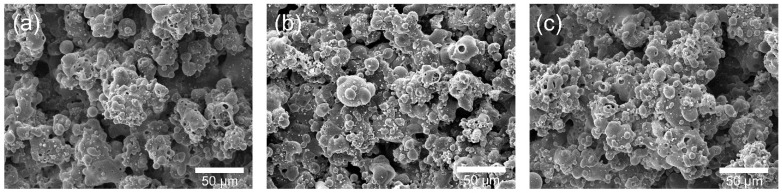
SEM images of HA/TiO_2_–PAM hydrogel–coated substrates: (**a**) before immersion, (**b**) after immersion in SBF for 7 days, and (**c**) after immersion in SBF for 14 days.

When this CaCO_3_-rich coating was later immersed in SBF (Figure 6b,c), the SEM evolution showed dense coverage by sub-micron globular deposits rather than the flower-like apatite crystals typically reported for conventional HA coatings. This difference may be related to two factors acting in combination. First, the partial transformation of HA into CaCO_3_ during spraying could have altered the mineralization pathway. Owing to its higher solubility compared to CaP phases, CaCO_3_ may accelerate Ca^2+^ release and thus promote rapid yet more homogeneous Ca–P precipitation, rather than the formation of discrete flower-like crystallites [38]. Indeed, previous studies have reported that carbonated HA particles and CaCO_3_-containing composites immersed in SBF tend to form continuous Ca–P layers instead of bouquet-like structures [39,40]. Second, the hydrogel texture may have contributed to this outcome by providing a hydrated, ion-permeable network that possibly modulated ion transport and nucleation. Such a matrix could help redistribute Ca^2+^ and PO_4_^3−^ ions, encouraging uniform deposition while suppressing localized supersaturation that typically gives rise to flower-like apatite.

**Figure 7:**
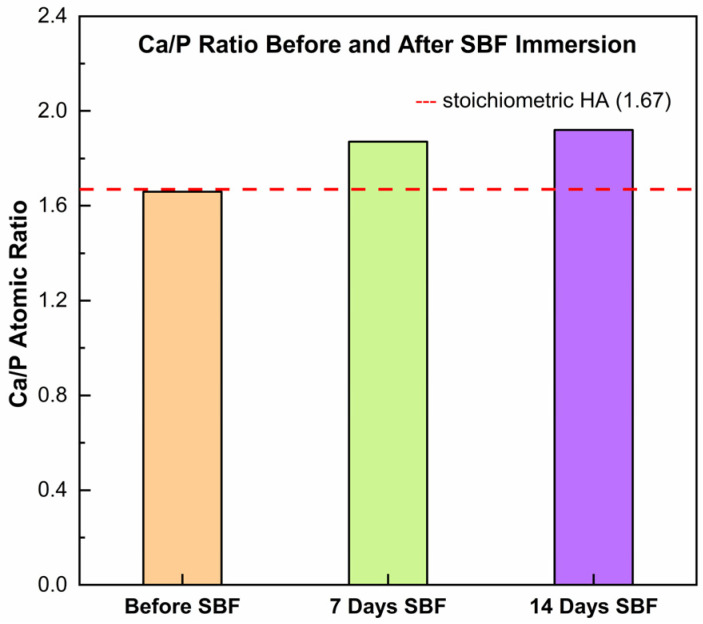
Evolution of the Ca/P atomic ratio in the HA/TiO_2_–hydrogel coating before and after immersion in simulated body fluid (SBF).

The Ca/P atomic ratio obtained by EDS (Figure 7) increased from ~1.66 in the as-sprayed coating to ~1.87 after 7 days in SBF and ~1.92 after 14 days. This progressive Ca enrichment indicates that the newly deposited phases were Ca-rich and not stoichiometric HA. One possible explanation is that carbonate present in the coating dissolved during immersion, supplying excess Ca^2+^ relative to phosphate uptake from the SBF. The released calcium then contributed to rapid precipitation of Ca-rich deposits (e.g., carbonated apatite or mixed CaCO_3_/Ca–P), which would shift the surface composition toward higher Ca/P values. Thus, the rising Ca/P ratio measured by EDS may reflect a carbonate-driven mineralization pathway, in which dissolution–reprecipitation processes favor the accumulation of Ca-rich layers at the coating surface under SBF conditions.

## 3. Conclusions

Flame-sprayed HA/TiO_2_ coatings with in situ hydrogel growth produced hybrid surfaces with potential for implant applications. Incorporation of 1% TiO_2_ supported uniform hydrogel formation and coating stability, while higher TiO_2_ contents led to excessive growth and partial delamination. The presence of CaCO_3_, a more soluble phase than HA, may facilitate Ca^2+^ release and contribute to controlled degradability and Ca–P mineralization. These findings demonstrate a feasible coating strategy that integrates flame-sprayed ceramics with photocatalytic hydrogel growth and shows favorable mineralization behavior in SBF.

## 4. Materials and Methods

### 4.1. Preparation of HA/TiO_2_

Nanohydroxyapatite (nHA) powders were synthesized following our previously reported procedure [41]. Briefly, 0.25 mol of calcium nitrate tetrahydrate (Ca(NO_3_)_2_·4H_2_O, Sigma Aldrich, St. Louis, MO, USA) was dissolved in dilute hydrogen peroxide (Merck, Rahway, NJ, USA) under continuous stirring, after which phosphoric acid (H_3_PO_4_, RCL Labscan, Bangkok, Thailand) was added dropwise to the calcium solution. The pH was adjusted to 10 by gradual addition of ammonium hydroxide (NH_4_OH, J.T. Baker, Phillipsburg, NJ, USA), resulting in the precipitation of white calcium phosphate. The precipitate was filtered, washed repeatedly with distilled water to remove residual ions, and calcined at 600 °C for 3 h to obtain crystalline nHA powders. HA/TiO_2_ composites were then prepared by mixing the synthesized nHA with anatase TiO_2_ (Sigma Aldrich) at 1 wt% and 5 wt%. The mixtures were ball-milled for 3 h and subsequently calcined at 400 °C for 2 h.

### 4.2. Substrate Preparation and Flame Spray Coating

Commercial 316L stainless steel was selected as the substrate material. Prior to coating, the surface was mechanically roughened using sandblasting to enhance adhesion between the coating and the metallic substrate. The HA/TiO_2_ powders obtained in Section 4.1 were then used for thermal spraying.

HA/TiO_2_ coatings were deposited using a MEC Powderjet-86 flame-spray gun, with the parameters summarized in Table 1. The powders were fed into an oxy-acetylene flame, propelled by nitrogen as carrier gas, and sprayed onto the pretreated stainless steel substrates. In addition to substrate coating, in-flight particles were also collected by directing the sprayed powders into a water bath placed opposite to the spray path. The sudden quenching preserved the particle morphology and phase composition during spraying, enabling comparison between the powders and the final coatings.

Preliminary optimization trials were carried out by varying the spray distance (170, 150, 120, and 100 mm) and using different nozzle types. For pure HA, optimal deposition required a longer spray distance of 170 mm with a K-type nozzle (multiple small peripheral orifices), which promoted adequate melting. In contrast, the addition of TiO_2_ increased the melting difficulty and promoted powder agglomeration, which made stable spraying more challenging. To overcome this, an M-type nozzle with a larger central orifice (designated P7C-M in this study) provided a more stable plume and uniform coatings. Among the tested conditions, 120 mm with the P7C-M nozzle yielded the most adherent and homogeneous HA/TiO_2_ coatings, and was therefore selected as the standard condition for this work.

### 4.3. Hydrogel Formation on HA/TiO_2_-Coated Stainless Steel via Self-Initiated Photopolymerization

Hydrogel growth was carried out via self-initiated photopolymerization, using TiO_2_ embedded within the HA/TiO_2_-coated stainless steel plates as photocatalyst. Acrylamide (AM, 2.0 M) was employed as the monomer, and N,N′-methylenebisacrylamide (NMBA, 0.16 M) was used as the crosslinker, corresponding to an AM:NMBA molar ratio of approximately 12.5:1. The precursors were dissolved in 500 mL of deionized water, and the coated plate was immersed in the solution and exposed to UVA irradiation for 3 h, enabling in situ polymerization and hydrogel formation on the substrate surface.

### 4.4. Characterization of Powders and Coatings

The crystalline phases of both the synthesized powders and the as-sprayed coatings were analyzed by X-ray diffraction (XRD, Rigaku SmartLab, Tokyo, Japan). Surface and cross-sectional morphologies were observed using scanning electron microscopy (SEM, TESCAN VEGA 3), while elemental composition was confirmed by energy-dispersive spectroscopy (EDS). Bioactivity was evaluated by immersing the coated substrates in simulated body fluid (SBF), prepared according to Kokubo’s protocol, for 7 and 14 days. After immersion, the surfaces were examined for apatite formation and morphological changes using SEM and EDS.

## Figures and Tables

**Table 1:** Thermal spray parameters for HA/TiO_2_ coatings.

Spraying Parameter	Value
Acetylene flow rate (L·min^−1^)	26
Oxygen flow rate (L·min^−1^)	21
Nitrogen flow rate (L·min^−1^)	1.6
Deposition rate (g·min^−1^)	3.95
Gun traverse speed (mm·s^−1^)	3.0
Step distance (cm)	1.0
Spray distance (mm)	120
Nozzle type	P7C-M

## Data Availability

The original contributions presented in this study are included in the article/Supplementary Material. Further inquiries can be directed to the corresponding author(s).

## Supplementary Materials

The following supporting information can be downloaded at: https://www.mdpi.com/article/10.3390/gels11100837/s1, Figure S1: Cross-sectional SEM images of the as-sprayed HA/TiO_2_ coating from independent trials; Figure S2: FTIR spectra of (a) HA/TiO_2_ powders and (b) hydrogel/HA/TiO_2_ coating; Figure S3: SEM-EDS analysis of the initial stage of in situ photopolymerization (after 1 h of UV irradiation); Figure S4: Swelling ratio (%) of the hydrogel/HA/TiO_2_ coating.
