# Supplementary material for: In Situ Hydrogel Growth on Flame-Sprayed Hydroxyapatite (HA)/TiO2-Coated Stainless Steel via TiO2-Photoinitiated Polymerization
Source: Gels. 2025 Oct 18;11(10):837. doi: 10.3390/gels11100837 (PMC12564610; doi:10.3390/gels11100837)
Supplement: Supplementary file 1 [file gels-11-00837-s001.zip › gels-3900204-supplementary.pdf]

Supplementary Material

In Situ Hydrogel Growth on Flame-Sprayed Hydroxyapatite (HA)/TiO_2-Coated Stainless Steel via TiO_2-Photoinitiated Polymerization

Komsanti Chokethawai [1], Nattawit Yutimit [1], Burin Boonsri [2], Parkpoom Jarupoom [3,4] , Ketmanee Muangchan [5], Sahadsawat Tonkaew [5], Pongpen Kaewdee [5,6], Sujitra Tandorn [5,6] and Chamnan Randorn [5,7,*]

[1]Department of Physics and Materials Science, Faculty of Science, Chiang Mai University, Chiang Mai 50200, Thailand

[2]Faculty of Veterinary Medicine, Chiang Mai University, Chiang Mai 50200, Thailand.

[3]Department of Industrial Engineering, Faculty of Engineering, Rajamangala University of Technology Lanna (RMUTL), Chiang Mai 50300, Thailand.

[4]Materials and Medical Innovation Research Unit, Faculty of Engineering, Rajamangala University of Technology Lanna (RMUTL), Chiang Mai 50300, Thailand

[5]Department of Chemistry, Faculty of Science, Chiang Mai University, Chiang Mai 50200, Thailand

[6]Office of Research Administration, Chiang Mai University, Chiang Mai 50200, Thailand

[7]Center of Excellence in Materials Science and Technology, Chiang Mai University, Chiang Mai 50200, Thailand

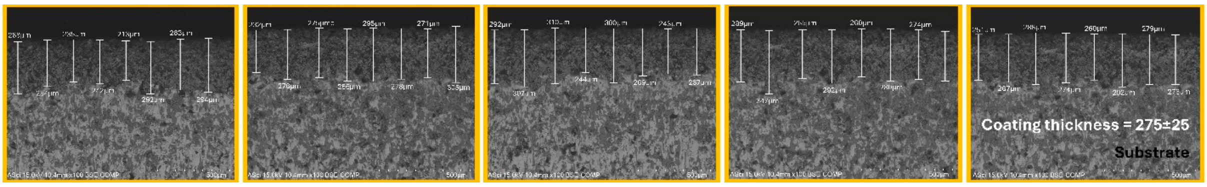

Figure S1. Cross-sectional SEM images of the as-sprayed HA/TiO_2 coatings from several independent trials

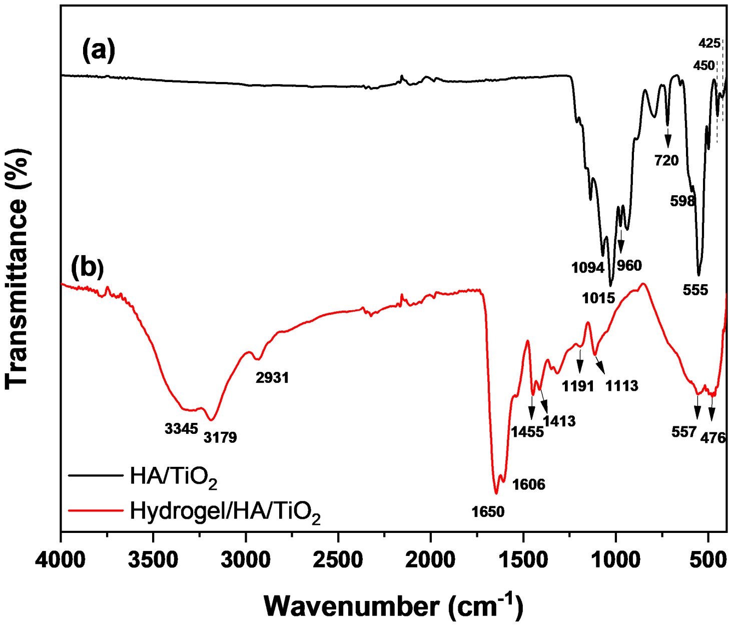

Figure S2. FTIR spectra of (a) HA/TiO_2 powders and (b) hydrogel/HA/TiO_2 coating

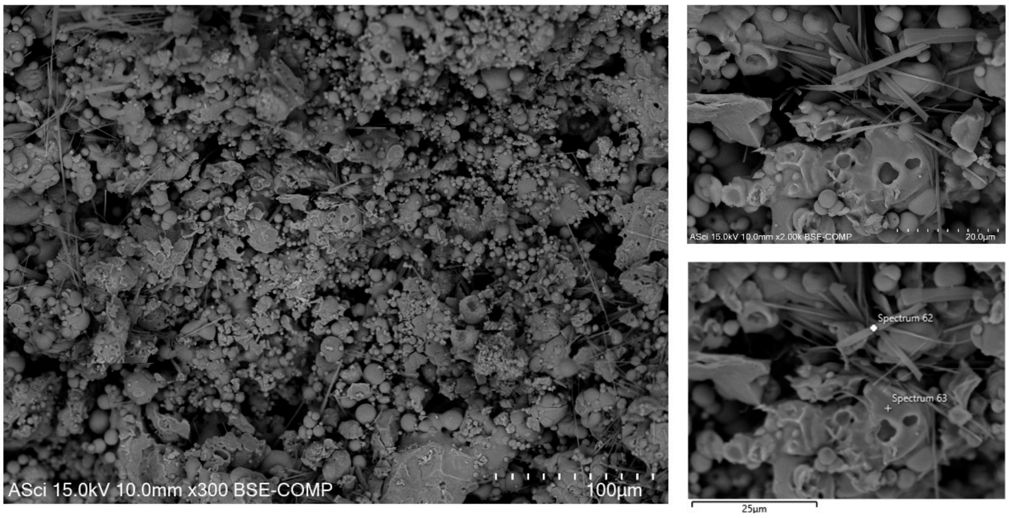

Spectrum 62

Element	Wt%
C	23.3
N	13.74
O	21.12
P	3.25
Ca	37.4
Ti	1.18
Total	100

Spectrum 63

Element	Wt%
C	4.01
O	18.4
P	21.79
Ca	55.17
Ti	0.62
Total	100

Figure S3. SEM–EDS analysis of the initial stage of in situ photopolymerization (after 1 h of UV irradiation)

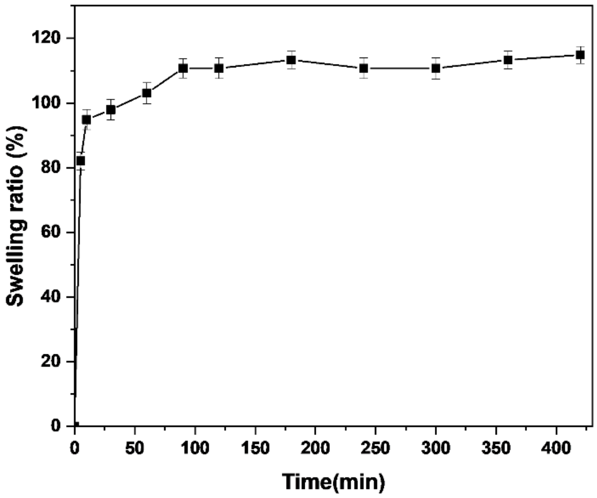

Figure S4. Swelling ratio (%) of the hydrogel/HA/TiO_2 coating
